# Cernunnos/XLF Deficiency: A Syndromic Primary Immunodeficiency

**DOI:** 10.1155/2014/614238

**Published:** 2014-01-08

**Authors:** Funda Erol Çipe, Cigdem Aydogmus, Arzu Babayigit Hocaoglu, Merve Kilic, Gul Demet Kaya, Elif Yilmaz Gulec

**Affiliations:** ^1^Department of Pediatric Allergy-Immunology, Kanuni Sultan Suleyman Research and Training Hospital, 34303 Istanbul, Turkey; ^2^Department of Genetics, Kanuni Sultan Suleyman Research and Training Hospital, 34303 Istanbul, Turkey

## Abstract

Artemis, DNA ligase IV, DNA protein kinase catalytic subunit, and Cernunnos/XLF genes in nonhomologous end joining pathways of DNA repair mechanisms have been identified as responsible for radiosensitive SCID. Here, we present a 3-year-old girl patient with severe growth retardation, bird-like face, recurrent perianal abscess, pancytopenia, and polydactyly. Firstly, she was thought as Fanconi anemia and spontaneous DNA breaks were seen on chromosomal analysis. After that DEB test was found to be normal and Fanconi anemia was excluded. Because of that she had low IgG and IgA levels, normal IgM level, and absence of B cells in peripheral blood; she was considered as primary immunodeficiency, Nijmegen breakage syndrome. A mutation in NBS1 gene was not found; then Cernunnos/XLF deficiency was investigated due to clinical similarities with previously reported cases. Homozygous mutation in Cernunnos/XLF gene (NHEJ1) was identified. She is now on regular IVIG prophylaxis and has no new infection. Fully matched donor screening is in progress for bone marrow transplantation which is curative treatment of the disease. In conclusion, the patients with microcephaly, bird-like face, and severe growth retardation should be evaluated for hypogammaglobulinemia and primary immunodeficiency diseases.

## 1. Introduction

Severe combined immunodeficiency (SCID) is defined as a group of disorders that affect both humoral and cellular immunity. Two groups of SCID have been defined: B(+) SCID (with residual B cells) and B(−) SCID (with absence of B cells) [1]. Approximately 35% of all SCIDs are radiosensitive and 4 proteins and responsible genes (*DCLRE1C* gene for Artemis, *LIG4* gene for DNA ligase IV, *PRKDC* gene for DNA protein kinase catalytic subunit, and *NHEJ1* gene for Cernunnos) in nonhomologous end joining pathways have been identified as responsible genes [1, 2]. DNA ligase IV and Cernunnos deficiency lead to microcephaly and, additionally, severe growth retardation [2].

Here, we present a 3-year-old girl admitted with severe growth retardation, recurrent perianal abscess, polydactyly, and pancytopenia. Low immunoglobulin (Ig)G, IgA levels, normal IgM levels, and agammaglobulinemia were detected and Cernunnos/XLF gene mutation was subsequently identified.

## 2. A Case Report

**Figure 1:**
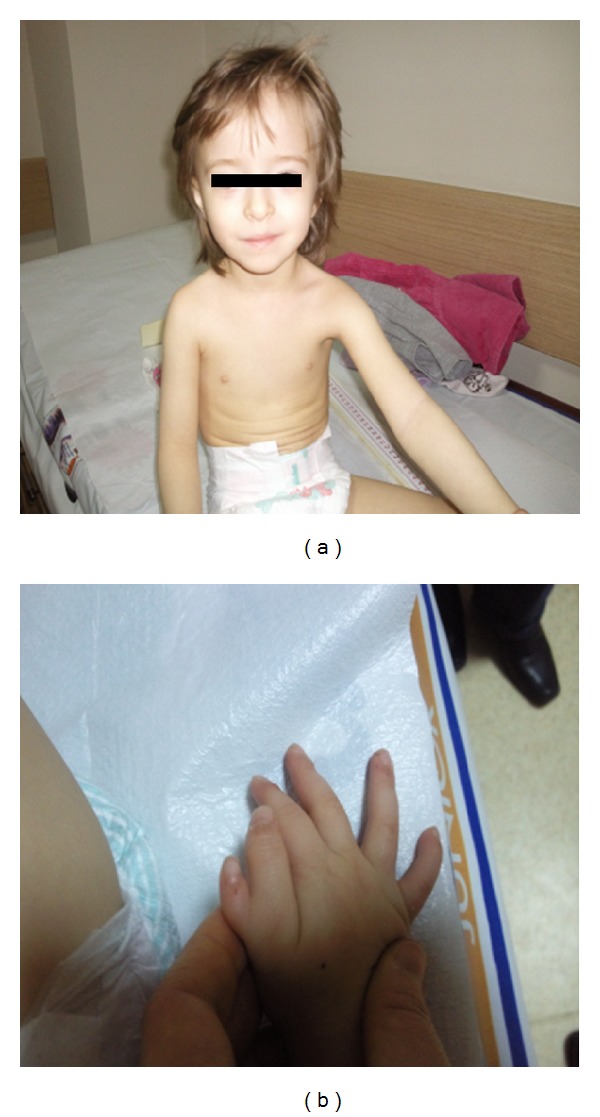
(a) Bird-like face, severe growth failure. (b) Preaxial polydactyly on her right hand.

**Figure 2:**
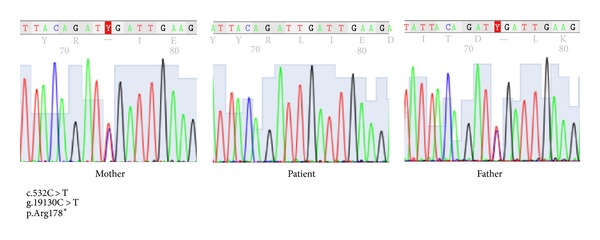
Mutation in *NHEJ1 *(Cernunnos) gene.

Three-year-old girl was transferred to our clinic for consultation because of growth retardation, pancytopenia, and recurrent perianal abscess. Her parents were consanguineous and two of her mother's siblings had died because of infections in early infancy without definitive diagnosis in her family history. On physical examination, she had severe growth failure (weight: 7200 g (<3rd percentile), height: 80 cm (<3rd percentile)), a bird-like facial appearance, microcephaly (46 cm, <3rd percentile), and polydactyly on her right hand (Figure 1). In laboratory investigations, prominent neutropenia (500–1500/mm^3^) and low Ig levels (IgG: 140 mg/dL, IgA: 22.6 mg/dL, and IgM: 84 mg/dL) were found. Her absolute lymphocyte count was normal and the percentage of CD3^+^ cells was 91% in the peripheral blood. But CD19^+^ and CD20^+^ cells were found to be absent. She was treated with intravenous immunoglobulin (IVIG) because of agammaglobulinemia. After IVIG treatment, her neutropenia improved and she had no new infection, but her lymphocyte counts decreased progressively to 600/*μ*L. Peripheral lymphocyte subsets were reanalyzed and found to be as follows: CD3^+^: 83%, CD4^+^: 42%, CD8^+^: 47%, CD19^+^ and CD20^+^: 0%, and CD16^+^56^+^: 17%. Genetic analysis showed spontaneous chromosomal breakages. Because she had pancytopenia, microcephaly, and polydactyly, Fanconi anemia was excluded with normal diepoxybutane (DEB) test. With these findings, Nijmegen breakage syndrome was firstly thought to be the diagnosis of the case but *NBS1 *mutation was found to be negative. She was still 80 cm (<3rd percentile) in height and 7200 gr (<3rd percentile) in weight at three years of age. As she had severe growth retardation, microcephaly, and mild combined immunodeficiency, the case was evaluated for Cernunnos/XLF deficiency and DNA ligase IV deficiency. After obtaining informed consent, we conducted a genetic analysis on DNA samples from the whole blood of the patient and a homozygous g.14130C > T,c.532C > T p.Arg178Ter mutation in the *NHEJ1* (Cernunnos) gene was found (Figure 2). Now that the diagnosis has been established, she is being prepared for bone marrow transplantation for curative treatment of SCID. A bone marrow transplantation will be conducted when a fully matched donor from either family or an unrelated donor bank is found.

## 3. Discussion

V(D)J recombination is a somatic rearrangement of Ig and T cell receptor genes for diversity of lymphocyte antigen receptors. The first step in V(D)J recombination is the generation of double-stranded DNA breaks (DSB). Subsequently, a hairpin formation is performed by the recombination of activating genes (RAG) 1 and 2 [2, 3]. Once DSBs are created by RAG complex, DNA repair must take place to properly differentiate, especially from Ig*μ* (−) to Ig*μ* (+) pre-B cells and from pro-T cells to double-negative pro-T cells [4].

There are two main repair pathways: nonhomologous end joining (NHEJ) and homologous recombination (HR). Defects in the genes of the HR pathway result in diseases such as ataxia telangiectasia, Seckel syndrome, Nijmegen breakage syndrome, and Fanconi anemia. These diseases are characterized by some physical abnormalities with some degree of immunodeficiency and cancer predisposition [4].

The NHEJ pathway is especially critical in the repair of DSBs during V(D)J recombination in T and B lymphocytes. The NHEJ pathway begins with the detection of DSB by Ku. After this step, NHEJ includes 3 major steps: synapsis by DNA-dependent protein kinase (DNA-PK), end processing by nucleases such as Artemis, and ligation carried out by DNA ligase IV, which is complexed with X-ray cross-complementation group 4 (XRCC4). XRCC4-like factor (XLF)/Cernunnos interacts with XRCC4 and induces the ligation by DNA ligase IV [3, 4].

Defects in any of the proteins in the NHEJ pathway result in the disruption of normal maturation of both T and B cells. Since NK cells do not undergo V(D)J recombination, patients have a T(−)B(−) NK(+) SCID phenotype. So far to date, mutations in the genes of *DCLRE1C*, *LIG4*, *PRKDC*, and *NHEJ1* have all been reported as causes of SCID in NHEJ pathway [5]. However, microcephaly and growth retardation are common in DNA ligase IV and Cernunnos/XLF deficiency not associated with Artemis and DNA-PKcs defects [6].

Cernunnos deficiency, causing SCID, was first described in 2006 [5, 6]. In the literature, 13 cases were reported (Turkish, Italian, and French); all had similarly low T cells and absent or very low B cell numbers with normal NK cells. Ig levels showed generally low IgG and IgA levels and normal IgM levels suggested the defect of class-switch recombination (CSR). Microcephaly, severe growth failure, and bird-like facial appearance were reported in most of the patients and two of them had autoimmune cytopenia. With the exception of two, they had mild combined immunodeficiency with recurrent bacterial and opportunistic infections [6–10]. Polydactyly, as in our case, had not been reported until now. However, we cannot say that it is due to Cernunnos deficiency because the parents are consanguineous, meaning that there are many mutated genes causing polydactyly.

The presented case was hospitalized firstly for pancytopenia, growth retardation, and perianal abscess. Because she had microcephaly, growth retardation, polydactyly, and bird-like face, she was firstly considered as Fanconi anemia. Supporting this diagnosis, spontaneous DNA breaks were seen in chromosomal analysis of the blood sample, which was conducted after informed consent for genetic analysis was obtained. With the detection of very low IgG and IgA levels and the absence of B lymphocytes she was thought to have Nijmegen breakage syndrome, which is a syndromic primary immunodeficiency mimicking Fanconi anemia. *NBS1* gene analysis was found to be negative; therefore, it was thought to be a different diagnosis. Finally, because of phenotypical and laboratory similarities with previously reported Cernunnos deficient patients, genetic analysis for Cernunnos/DNA ligase IV deficiency was performed and homozygous *NHEJ1* (Cernunnos) mutation, which is common in Turkish patients, was identified [7, 8].

IgM levels in this case were elevated at the time of diagnosis as in most reported patients. During IVIG replacement treatment, IgM levels decreased to normal levels. High IgM levels caused us to consider that Cernunnos might play a role in CSR, in addition to V(D)J recombination in the literature [3].

Our patient firstly presented with pancytopenia. After IVIG replacement, neutropenia improved but lymphopenia developed. Although we could not show autoantibodies, we thought that pancytopenia might be autoimmune or secondary to the existent infection.

She is now being followed up with IVIG treatment and a fully matched donor is being sought for bone marrow transplantation. In one report, two patients have received hematopoietic stem cells from an HLA identical related donor without conditioning regimen and recovered without any complication [10].

In conclusion, the patients with microcephaly, bird-like face, severe growth retardation, and hypogammaglobulinemia should be evaluated for DNA repair defects like Nijmegen breakage syndrome. Although Fanconi aplastic anemia is generally the first possible diagnosis in patients with pancytopenia and microcephaly, Ig levels and lymphocyte subtypes must be evaluated in the patients especially recurrent severe infections. Mutation analysis of these patients will help to identify genetic defects especially in other components of DNA repair mechanisms.
